# Llamamiento a adoptar medidas urgentes para limitar los aumentos de temperatura en el mundo, restablecer la diversidad biológica y proteger la salud^*^: Las naciones ricas deben hacer mucho más y con mayor rapidez

**DOI:** 10.26633/RPSP.2021.123

**Published:** 2021-09-07

**Authors:** Lukoye Atwoli, Abdullah H. Baqui, Thomas Benfield, Raffaella Bosurgi, Fiona Godlee, Stephen Hancocks, Richard Horton, Laurie Laybourn-Langton, Carlos Augusto Monteiro, Ian Norman, Kirsten Patrick, Nigel Praities, Marcel GM Olde Rikkert, Eric J. Rubin, Peush Sahni, Richard Smith, Nick Talley, Sue Turale, Damián Vázquez

**Affiliations:** 1 Editor jefe, East African Medical Journal Editor jefe, East African Medical Journal; 2 Editor jefe, Journal of Health, Population and Nutrition Editor jefe, Journal of Health, Population and Nutrition; 3 Editor jefe, Danish Medical Journal Editor jefe, Danish Medical Journal; 4 Editor jefe, PLOS Medicine Editor jefe, PLOS Medicine; 5 Editor jefe, The BMJ Editor jefe, The BMJ; 6 Editor jefe, British Dental Journal Editor jefe, British Dental Journal; 7 Editor jefe, The Lancet Editor jefe, The Lancet; 8 Asesor principal, UK Health Alliance on Climate Change Asesor principal, UK Health Alliance on Climate Change; 9 Editor jefe, Revista de Saúde Pública Editor jefe, Revista de Saúde Pública; 10 Editor jefe, International Journal of Nursing Studies Editor jefe, International Journal of Nursing Studies; 11 Editor jefe interino, CMAJ Editor jefe interino, CMAJ; 12 Editor ejecutivo, Pharmaceutical Journal Editor ejecutivo, Pharmaceutical Journal; 13 Editor jefe, Dutch Journal of Medicine Editor jefe, Dutch Journal of Medicine; 14 Editor jefe, NEJM Editor jefe, NEJM; 15 Editor jefe, National Medical Journal of India Editor jefe, National Medical Journal of India; 16 Presidente, UK Health Alliance on Climate Change Presidente, UK Health Alliance on Climate Change; 17 Editor jefe, Medical Journal of Australia Editor jefe, Medical Journal of Australia; 18 Editor jefe, International Nursing Review Editor jefe, International Nursing Review; 19 Editor jefe, Revista Panamericana de Salud Pública. Editor jefe, Revista Panamericana de Salud Pública.

En septiembre del 2021, la Asamblea General de las Naciones Unidas reunirá a los países en un momento crucial para organizar la acción colectiva con el propósito de hacer frente a la crisis medioambiental mundial. Se reunirán una vez más en la Conferencia de las Naciones Unidas sobre la Diversidad Biológica, en Kunming (China) y en la Conferencia de las Naciones Unidas sobre el Cambio Climático (CP26), en Glasgow (Escocia). Antes de la celebración de estas reuniones trascendentales, nosotros —los editores de revistas sobre salud de todo el mundo— exigimos medidas urgentes para mantener los aumentos promedio de la temperatura a nivel mundial por debajo de 1,5 °C, detener la destrucción de la naturaleza y proteger la salud.

La salud ya se está deteriorando debido a los aumentos de temperatura a nivel mundial y a la destrucción del mundo natural, una situación que los profesionales de la salud han venido mencionando desde hace varios decenios.^1^ La ciencia es inequívoca; un aumento mundial de 1,5 °C por encima del promedio preindustrial y la pérdida continua de la diversidad biológica representan un riesgo de daño catastrófico para la salud que será imposible de revertir.^2^^,^^3^ A pesar de la preocupación inevitable en todo el mundo causada por la COVID-19, no podemos esperar a que pase la pandemia para reducir rápidamente las emisiones.

Este editorial, en el que se plantea la gravedad del momento, se publica en varias revistas de salud de todo el mundo. Estamos unidos en el reconocimiento de que nuestra trayectoria actual solo se revertirá mediante cambios fundamentales y equitativos en la sociedad.

Los riesgos para la salud de los aumentos de la temperatura superiores a 1,5 °C ya son bien conocidos.^2^ En efecto, ningún aumento de la temperatura es “seguro”. En los últimos 20 años, la mortalidad de personas de más de 65 años por causa del calor ha subido en más de 50%.^4^ Las temperaturas más altas han dado lugar al aumento de la deshidratación y a la insuficiencia renal, a neoplasias de la piel, a infecciones tropicales, a resultados adversos de salud mental, a complicaciones del embarazo, a alergias, así como a morbilidad y mortalidad por problemas cardiovasculares y pulmonares.^5^^,^^6^ Los daños afectan desproporcionadamente a los más vulnerables, como los niños, los adultos mayores, las minorías étnicas, las comunidades más pobres y quienes tienen problemas de salud subyacentes.^2^^,^^4^

El calentamiento en todo el mundo es además uno de los factores responsables de la disminución del rendimiento potencial de los principales cultivos a nivel mundial, el cual se ha reducido entre 1,8% y 5,6% desde 1981; esto, junto con los efectos del clima extremo y el agotamiento de los suelos, obstaculiza las iniciativas para reducir la desnutrición.^4^ La salud humana requiere ecosistemas que prosperen; la destrucción generalizada de la naturaleza, que abarca los hábitats y las especies, ha erosionado la seguridad del agua y de los alimentos y aumentado las probabilidades de que ocurran pandemias.^3^^,^^7^^,^^8^

Las consecuencias de la crisis ambiental recaen de manera desproporcionada en los países y las comunidades cuya contribución al problema ha sido mínima y cuya capacidad para mitigar los daños es mucho menor. Sin embargo, ningún país, por muy rico que sea, podrá estar a salvo de estas repercusiones. Si se permite que las consecuencias recaigan en forma desproporcionada sobre los más vulnerables, aumentarán los conflictos, la inseguridad alimentaria, los desplazamientos forzados y las zoonosis, con consecuencias graves para todos los países y comunidades. Del mismo modo que sucede con la pandemia de COVID-19, a nivel mundial somos tan fuertes como nuestro miembro más débil.

Los aumentos de temperatura superiores a 1,5 °C incrementan las probabilidades de llegar a los puntos de inflexión en los sistemas naturales que podrían colocar al mundo en una situación sumamente inestable. Esto perjudicaría de manera crucial nuestra capacidad de mitigar los daños y de evitar cambios ambientales catastróficos e incontrolados.^9^^,^^10^

## LAS METAS MUNDIALES NO BASTAN

Es alentador que muchos gobiernos, instituciones financieras y empresas estén fijando metas para alcanzar la cifra de cero emisiones netas, incluidas metas para el 2030. El costo de la energía renovable está disminuyendo de manera acelerada. Muchos países buscan proteger por lo menos 30% de la tierra y los océanos del planeta para el 2030.^11^

No basta hacer estas promesas. Las metas son fáciles de fijar y difíciles de lograr. Todavía tienen que armonizarse con planes creíbles de corto y más largo plazo para acelerar el uso de tecnologías menos contaminantes y transformar la sociedad. Los planes de reducción de las emisiones no incorporan de manera apropiada las consideraciones relativas a la salud.^12^ Preocupa cada vez más que los miembros poderosos de la comunidad mundial empiecen a considerar inevitable, o hasta aceptable, que la temperatura suba por encima de 1,5 °C.^13^ En relación con lo anterior, las estrategias actuales para reducir a cero las emisiones netas hacia mediados del siglo suponen, de manera poco probable, que el mundo logrará incrementar considerablemente su capacidad para extraer los gases de efecto invernadero de la atmósfera.^14^^,^^15^

Esta falta de acción implica que es probable que los aumentos de temperatura sean bastante más altos que los 2 °C,^16^ un resultado catastrófico para la estabilidad de la salud y el medioambiente. Hay un factor clave: la destrucción de la naturaleza no tiene la misma importancia que el elemento climático de la crisis; no se ha alcanzado ninguna de las metas mundiales para restaurar la pérdida de diversidad biológica en el 2020.^17^ Esta es una crisis medioambiental general.^18^

Los profesionales de salud están colaborando con científicos ambientales, empresas y muchos otros para rechazar la idea de que este resultado es inevitable. Hay que hacer más y hay que hacerlo ya, en Glasgow, en Kunming y en los años inmediatamente posteriores. Nos unimos a los profesionales de salud de todo el mundo, quienes ya han apoyado los llamamientos en pro de una acción acelerada.^19^^,^^1^

La equidad debe ser fundamental en la respuesta mundial. Aportar una proporción justa al esfuerzo mundial significa que los compromisos de reducción deben tener en cuenta la contribución acumulativa e histórica que cada país ha hecho a las emisiones, así como sus emisiones actuales y su capacidad de respuesta. Los países más ricos tendrán que reducir las emisiones más rápidamente, lograr para el 2030 reducciones superiores a las que se han propuesto en la actualidad^20^^,^ ^21^ y alcanzar cero emisiones netas antes del 2050. Es necesario fijar metas y adoptar medidas de emergencia similares con respecto a la pérdida de la diversidad biológica y al aumento de la destrucción del mundo natural.

Para lograr estas metas, los gobiernos deben hacer cambios fundamentales en la manera en que se organiza la economía y la sociedad, así como en la manera en que vivimos. La estrategia actual de alentar a los mercados para que cambien las tecnologías contaminantes por tecnologías más limpias no es suficiente. Los gobiernos deben intervenir para apoyar el rediseño de los sistemas de transporte, las ciudades, la producción y distribución de alimentos, los mercados para las inversiones financieras, los sistemas de salud y mucho más. Se necesita la coordinación mundial para que la prisa por adoptar tecnologías más limpias no conduzca al aumento de la destrucción medioambiental ni a la explotación humana.

Muchos gobiernos hicieron frente a la amenaza de la pandemia de COVID-19 con un financiamiento nunca visto. La crisis medioambiental exige una respuesta similar de emergencia. Hará falta una inversión gigantesca, más allá de lo que se está considerando o ejecutando en cualquier lugar del mundo. Sin embargo, esas inversiones van a generar resultados enormes y positivos tanto para la economía como para la salud, entre los cuales se encuentran empleos de buena calidad, una reducción de la contaminación del aire, un aumento de la actividad física y el mejoramiento de la vivienda y la alimentación. El mejoramiento de la calidad del aire por sí solo lograría beneficios para la salud que compensan fácilmente los costos mundiales de reducción de las emisiones.^22^

Estas medidas también mejorarán los determinantes sociales y económicos de la salud, cuya situación inadecuada podría haber contribuido a que ciertos grupos de la población fuesen más vulnerables que otros a la pandemia de COVID-19.^23^ Sin embargo, no es posible lograr cambios si se retorna a las políticas de austeridad perjudiciales o si persisten las grandes desigualdades de riqueza y poder que existen entre los países y dentro de ellos.

## LA COOPERACIÓN DEPENDE DE QUE LAS NACIONES RICAS HAGAN MÁS

En particular, los países que han contribuido de manera desproporcionada a la crisis medioambiental deben trabajar más a fin de apoyar a los países de ingresos bajos y medianos en la creación de sociedades más limpias, más sanas y más resilientes. Los países de ingresos altos deben cumplir y superar su compromiso pendiente de aportar US$ 100 mil millones anuales, para compensar cualquier insuficiencia ocurrida en el 2020, y aumentar sus contribuciones hasta el 2025 y los años posteriores. El financiamiento deberá distribuirse por igual entre la mitigación y la adaptación, lo que incluirá mejorar la resiliencia de los sistemas de salud.

El financiamiento deberá hacerse mediante subvenciones y no con préstamos, para crear capacidad local y empoderar realmente a las comunidades, lo cual deberá estar unido a la condonación de las deudas grandes, que limitan las acciones de tantos países de ingresos bajos. Deberá organizarse financiamiento adicional para compensar las pérdidas y los daños inevitables derivados de la crisis medioambiental.

En nuestra calidad de profesionales de la salud, debemos hacer todo lo que esté a nuestro alcance para apoyar la transición a un mundo sostenible, más justo, resiliente y con más salud. Además de participar en la reducción del daño ocasionado por la crisis medioambiental, debemos contribuir con dinamismo a la prevención de otros daños en todo el mundo y actuar sobre las causas originarias de la crisis. Debemos responsabilizar a los líderes mundiales y seguir concientizando a otros acerca de los riesgos para la salud resultantes de la crisis. Debemos participar en la tarea de establecer sistemas de salud sostenibles desde el punto de vista medioambiental antes del 2040, sin dejar de reconocer que esto significará cambiar la práctica clínica. Las instituciones de salud ya han retirado más de $ 42 mil millones de los activos que habían invertido en combustibles fósiles; otros deben seguir este ejemplo.^4^

La mayor amenaza para la salud pública mundial es que los líderes mundiales sigan fracasando en los esfuerzos por mantener el aumento de la temperatura a nivel mundial por debajo de 1,5 °C y por restaurar la naturaleza. Deberán hacerse cambios urgentes que abarquen a toda la sociedad, ya que ellos conducirán a un mundo más justo y con mejor salud. Nosotros, como editores de las revistas de salud, hacemos un llamamiento a los gobiernos y a otros líderes para que actúen, lo cual marcará el 2021 como el año en el que, finalmente, el mundo cambió su curso.

## Declaración.

Este editorial se está publicando simultáneamente en numerosas revistas internacionales. Sírvase ver la lista completa aquí: https://www.bmj.com/content/full-list-authors-and-signatories-climate-emergency-editorial-september-2021

## References

[1] 1. In support of a health recovery. https://healthyrecovery.net

[2] 2. Intergovernmental Panel on Climate Change. Summary for policymakers. In: Global warming of 1.5°C. An IPCC special report on the impacts of global warming of 1.5°C above pre-industrial levels and related global greenhouse gas emission pathways, in the context of strengthening the global response to the threat of climate change, sustainable development, and efforts to eradicate poverty. 2018. https://www.ipcc.ch/sr15/

[3] 3. Intergovernmental Science-Policy Platform on Biodiversity and Ecosystem Services. Summary for policymakers: the global assessment report on biodiversity and ecosystem services. 2019. https://ipbes.net/sites/default/files/2020-02/ipbes_global_assessment_report_summary_for_policymakers_en.pdf

[4] 4. Watts N, Amann M, Arnell N, et al. The 2020 report of the Lancet Countdown on health and climate change: responding to converging crises. *Lancet* 2021;397:129-70. PubMed 10.1016/S0140-6736(20)32290-X PMC7616803 33278353

[5] 5. Rocque RJ, Beaudoin C, Ndjaboue R, et al. Health effects of climate change: an overview of systematic reviews. *BMJ Open* 2021;11:e046333. doi:10.1136/bmjopen-2020-046333 PubMed 10.1136/bmjopen-2020-046333 PMC8191619 34108165

[6] 6. Haines A, Ebi K. The imperative for climate action to protect health. *N Engl J Med* 2019;380:263-73. PubMed 10.1056/NEJMra1807873 30650330

[7] 7. United Nations Environment Programme and International Livestock Research Institute. *Preventing the next pandemic: zoonotic diseases and how to break the chain of transmission.* 2020.https://72d37324-5089-459c-8f70-271d19427cf2.filesusr.com/ugd/056cf4_b5b2fc067f094dd3b2250cda15c47acd.pdf

[8] 8. IPCC. 2019: Summary for policymakers. In: Climate change and land: an IPCC special report on climate change, desertification, land degradation, sustainable land management, food security, and greenhouse gas fluxes in terrestrial ecosystems. Forthcoming.

[9] 9. Lenton TM, Rockström J, Gaffney O, et al. Climate tipping points—too risky to bet against. *Nature* 2019;575:592-5. PubMed 10.1038/d41586-019-03595-0 31776487

[10] 10. Wunderling N, Donges JF, Kurths J, Winkelmann R. *Interacting tipping elements increase risk of climate domino effects under global warming.* Earth System Dynamics Discussions, 2020: 1-21.

[11] 11. High Ambition Coalition. https://www.hacfornatureandpeople.org

[12] 12. Global Climate and Health Alliance. Are national climate commitments enough to protect our health? https://climateandhealthalliance.org/initiatives/healthy-ndcs/ndc-scorecards/

[13] 13. Climate strikers: Open letter to EU leaders on why their new climate law is ‘surrender.’ Carbon Brief 2020. https://www.carbonbrief.org/climate-strikers-open-letter-to-eu-leaders-on-why-their-new-climate-law-is-surrender

[14] 14. Fajardy M, Köberle A, MacDowell N, Fantuzzi A. “BECCS deployment: a reality check.” Grantham Institute briefing paper 28, 2019. https://www.imperial.ac.uk/media/imperial-college/grantham-institute/public/publications/briefing-papers/BECCS-deployment---a-reality-check.pdf

[15] 15. Anderson K, Peters G. The trouble with negative emissions. *Science* 2016;354:182-3. PubMed 10.1126/science.aah4567 27738161

[16] 16. Climate action tracker. https://climateactiontracker.org

[17] 17. Secretariat of the Convention on Biological Diversity. *Global biodiversity outlook 5.* 2020. https://www.cbd.int/gbo5

[18] 18. Steffen W, Richardson K, Rockström J, et al. Sustainability. Planetary boundaries: guiding human development on a changing planet. *Science* 2015;347:1259855. http://doi:10.1126/science.1259855 PubMed 10.1126/science.1259855 25592418

[19] 19. UK Health Alliance. Our calls for action. http://www.ukhealthalliance.org/cop26/

[20] 20. Climate Action Tracker. *Warming projections global update: May 2021*. https://climateactiontracker.org/documents/853/CAT_2021-05-04_Briefing_Global-Update_Climate-Summit-Momentum.pdf

[21] 21. United Nations Environment Programme. *Emissions gap report 2020.* UNEP, 2020.

[22] 22. Markandya A, Sampedro J, Smith SJ, et al. Health co-benefits from air pollution and mitigation costs of the Paris Agreement: a modelling study. *Lancet Planet Health* 2018;2:e126-33. http://doi:10.1016/S2542-5196(18)30029-9 PubMed 10.1016/S2542-5196(18)30029-9 29615227

[23] 23. Paremoer L, Nandi S, Serag H, Baum F. Covid-19 pandemic and the social determinants of health. *BMJ* 2021;372:n129. PubMed 10.1136/bmj.n129 PMC7842257 33509801

